# Characteristics and outcomes of patients with therapy-related acute myeloid leukemia with normal karyotype

**DOI:** 10.1038/s41408-020-0316-3

**Published:** 2020-05-04

**Authors:** Bachar Samra, Guillaume Richard-Carpentier, Tapan M. Kadia, Farhad Ravandi, Naval Daver, Courtney D. DiNardo, Ghayas C. Issa, Prithviraj Bose, Marina Y. Konopleva, Musa Yilmaz, Maro Ohanian, Gautam Borthakur, Guillermo Garcia-Manero, Sherry Pierce, Jorge E. Cortes, Hagop Kantarjian, Nicholas J. Short

**Affiliations:** 1 Department of Leukemia, The University of Texas MD Anderson Cancer Center, 1515 Holcombe Blvd, Houston, TX 77030 USA; 2 Georgia Cancer Center at Augusta University, 1411 Laney Walker Blvd, Augusta, GA 30912 USA

**Keywords:** Chemotherapy, Cancer genomics, Cancer, Acute myeloid leukaemia

## Abstract

Normal karyotype in therapy-related acute myeloid leukemia (t-AML) is rare and the relative contribution of prior exposure to chemotherapy or radiotherapy to outcomes in these patients remains uncertain. We performed a retrospective study of 742 patients with newly diagnosed AML and normal karyotype (t-AML, *n* = 61, and non-t-AML, *n* = 681). Patients with t-AML were older but had a similar mutational profile compared to those with non-t-AML. Overall survival (OS) and relapse-free survival (RFS) were significantly worse for patients with t-AML (*P* < 0.01 and *P* = 0.02, respectively). Patients with t-AML had a higher cumulative incidence of death in remission (51% versus 16%, *P* < 0.01), but not higher cumulative incidence of relapse (42% versus 56%, respectively, *P* = 0.21). Both intensive induction and allogeneic hematopoietic stem cell transplantation in first remission were associated with improved OS and RFS in non-t-AML but not in t-AML. Overall, although disease biology appears similar between t-AML and non-t-AML with normal karyotype as indicated by similar risks of relapse, death in remission is the main driver of inferior outcome in t-AML. Careful therapeutic decisions are required to mitigate potential treatment-related toxicity in this rare subgroup of patients with t-AML and normal karyotype.

## Introduction

Therapy-related acute myeloid leukemia (t-AML) is a distinct clinical entity recognized by the World Health Organization (WHO) as a late complication occurring after exposure to cytotoxic chemotherapy and/or radiation therapy^1^. t-AML accounts for approximately 5–20% of all AML cases, and outcomes are generally poorer compared with de novo AML^2–4^. Several patient-related or disease-related factors explain the poor prognosis of patients with t-AML. Patient-related factors include older age at presentation, higher number of comorbidities and sequelae from prior cancer and prior therapy. Disease-related factors include higher frequency of adverse risk features such as complex cytogenetics, chromosomal aneuploidies (5/5q, -7/7q-) and high frequency of *TP53* mutations, all associated with resistance to conventional cytotoxic chemotherapy^4–11^. In multiple reports, the adverse prognosis observed in patients with t-AML was not independent of other variables including age, cytogenetics and molecular features raising the question whether the presence of t-AML, per se, confers a poor prognosis versus prognosis influenced by its strong association with older age and adverse cytogenetics^4,12–15^. For instance, the dismal outcome observed among patients with t-AML in one large study (median OS of 8 months) was primarily driven by abnormalities of chromosome 5 and 7, whereas t-AML with favorable karyotype (core binding factor-AML) had a median OS of 26.7 months^4^. Several reports suggest that the prognosis of t-AML is likely similar to that of de novo AML with corresponding cytogenetic risk^13,15^.

Normal karyotype (NK) among patients with t-AML is rare, accounting for <20% of cases of t-AML^3,12,16–18^. Because of its rare occurrence, the molecular features and clinical outcomes of patients with t-AML and normal karyotype are undefined, and it remains unknown whether prior exposure to chemotherapy or radiotherapy in these patients is associated with more aggressive disease biology and higher risk of relapse. Therefore, we aimed to compare the clinical and molecular characteristics of t-AML and non-t-AML with NK and to determine the prognostic impact of prior chemotherapy or radiotherapy exposure in patients with AML and NK.

## Material and methods

### Patients

We reviewed the medical records of all patients with newly diagnosed AML with available cytogenetic information treated at our institution between January 2008 and May 2019. The diagnosis of AML was confirmed by evaluation of a bone marrow biopsy by an expert hemato-pathologist following the WHO 2008 criteria. We focused our analyses on patients with NK, separating them into two groups (t-AML and non-t-AML) based on their prior exposure to chemotherapy or radiotherapy, regardless of the latency period. We collected the patients’ baseline clinical and molecular characteristics and outcomes including remission status and occurrence of relapse and death, and the intensity of therapy received. Intensive induction chemotherapy was defined as any regimen containing an anthracycline and/or intermediate to high-dose cytarabine (defined as cumulative dose of cytarabine ≥700 mg/m^2^). Other regimens were considered low-intensity therapies (Supplemental Table 1).

### Cytogenetic and molecular analysis

Chromosome G-banding was performed using standard techniques, and karyotypes were described according to the International System for Human Cytogenetic Nomenclature. The ELN 2017 risk classification was used to categorize patients into risk groups based on their mutational status^19^. Next generation sequencing was not available on all patients as this became routine at our institution in 2013. After this time, 28- or 81-gene myeloid panels were performed on all patients with an analytical sensitivity of 5% or better, as previously described^20^. Mutational testing for *FLT3* and *CEBPA* were performed using separate PCR-based assays.

### Clinical outcomes and statistical analyses

Response criteria including complete remission (CR), complete remission with incomplete hematologic recovery (CRi), morphologic leukemia-free state (MLFS) and partial remission (PR) were defined according to the 2017 ELN criteria^19^. Descriptive statistics were calculated for baseline patients’ characteristics separately for patients with t-AML and non-t-AML. Variables were compared between the two groups using Chi-square or Fisher exact tests for categorical variables and Student’s t test for continuous variables. Relapse-free survival (RFS) was defined as the time from achieving CR/CRi to relapse, death or last follow-up. OS was defined as the time from start of therapy to death or last follow-up. The Kaplan-Meier method was used to estimate the probabilities for RFS and OS and differences between groups were evaluated with the log-rank test. The cumulative incidence of relapse (CIR) and cumulative incidence of death (CID) were defined as time from remission to relapse and death in remission, respectively, considered as competing events for failure. The Gray’s test was used to compare cumulative incidence probabilities between groups. Univariate and multivariate analyses for RFS and OS were performed using the Cox proportional hazards regression model to calculate hazard ratios (HR) and confidence intervals (CI). Allogeneic hematopoietic stem cell transplantation (HSCT) was considered a time-dependent covariate in the extension of the Cox model. Statistical analyses were performed with R statistical software (version 3.5.1). *P*-values <0.05 were used to define statistical significance.

## Results

### Patient characteristics

**Fig. 1:**
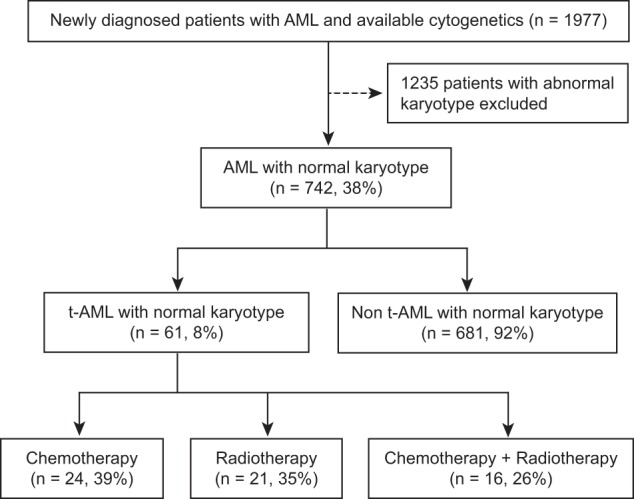
Schema of patient population. Among 1977 patients with AML who had available cytogenetics, 742 patients had normal karyotype (38% of the entire cohort). Among them, 61 patients (8%) had t-AML and 681 patients (92%) had non-t-AML.

A total of 1977 patients with AML and available cytogenetic information were identified including 340 patients with t-AML. Among 742 patients with NK (38% of the entire cohort), 61 patients (8%) had t-AML and 681 patients (92%) had non-t-AML (Fig. 1). NK was identified in 17% of all t-AML (61/340 patients). Prior therapy in patients with NK t-AML was chemotherapy alone (24 patients, 39%), radiotherapy alone (21 patients, 34%), or both (16 patients, 26%). Baseline characteristics of patients with NK, stratified by t-AML or non-t-AML, are summarized in Table 1. The median age was higher for patients with t-AML versus non-t-AML (71 years [range, 48–89] vs 64 years [range, 18–92], respectively, *P* < 0.01). Similar proportions of patients in both groups had secondary AML arising from antecedent hematologic disorder (10 and 12%, respectively, *P* = 0.63) (Supplemental Table 2). No statistically significant difference was noted in mutation frequencies or ELN 2017 risk categories. *NPM1* mutations were the most common genomic alteration in both groups (45% in t-AML versus 39% in non-t-AML, *P* = 0.38). Patients with t-AML had a higher frequency of *TP53* mutation (9% versus 4%) and a lower frequency of *CEBPA* mutations (5% versus 15%); however, these differences were not statistically significant (*P* = 0.14, and *P* = 0.07, respectively).

**Table 1:** Baseline characteristics of patients with AML with normal karyotype.

Characteristics	t-AML (*n* = 61)	Non-t-AML (*n* = 681)	*P*-value
	*N* (%)/median [range]	*N* (%)/median [range]	
**Age, years**	71 [48–89]	64 [18–92]	**<0.01**
Age ≥60 year-old	55 (90)	421 (62)	**<0.01**
**Hematological parameters**			
WBC count, ×10^9^/L	3.1 [0.2–106.8]	4.0 [0.2–378.4]	0.68
Hemoglobin, g/dl	9.3 [5.5–12.9]	9.3 [5.1–17.5]	0.81
Platelet count, ×10^9^/L	47 [7–271]	46 [1–584]	0.83
**Bone marrow blasts, %**	59 [4–95]	50 [0–98]	0.65
**ELN 2017 risk category**			
Favorable	10/30 (33.3)	71/337 (21)	0.12
Intermediate	10/30 (33.3)	129/337 (38)	0.59
Adverse	10/30 (33.3)	137/337 (41)	0.43
Insufficient molecular data available	31	344	
**Mutational status**			
*NPM1*	24/53 (45)	237/605 (39)	0.38
*DNMT3A*	10/36 (28)	138/408 (34)	0.46
*FLT3-*ITD	13/57 (23)	202/668 (30)	0.23
*RUNX1*	6/29 (21)	55/325 (17)	0.6
*ASXL1*	4/29 (14)	81/328 (25)	0.18
*TP53*	3/34 (9)	14/383 (4)	0.14
*FLT3-*TKD	5/57 (9)	43/668 (7)	0.91
*CEBPA*	2/42 (5)	73/491 (15)	0.07

*t-AML* therapy-related acute myeloid leukemia, *WBC* white blood cells, *ELN* European LeukemiaNet, *ITD* internal tandem duplication, *TKD* tyrosine kinase domain.

Bold values are statistically significant.

### Response rates and early mortality

Intensive induction chemotherapy was less frequently administered to patients with t-AML compared to those with non-t-AML (26% versus 52%, respectively, *P* < 0.01). However, the rates of HSCT in first remission (CR1) were similar between the two groups (15 and 22%, respectively*, P* = 0.17) as well as the rates of enrollment in clinical trials (80 and 84%, respectively, *P* = 0.45). Since induction therapy intensity markedly differed between groups and could influence response rates and survival outcomes, we stratified patients according to intensity of treatment (Table 2). In patients who received intensive chemotherapy, there was a trend for lower CR/CRi rates in patients with t-AML compared with non-t-AML (69% versus 86%, *P* = 0.05). Conversely, among patients who received low-intensity induction therapy, CR/CRi rates were similar among those with t-AML versus non-t-AML (60% versus 61%, *P* = 0.92). Of note, venetoclax-based therapy (most commonly, azacitidine or decitabine in combination with venetoclax) was used in 8 patients (13%) with t-AML and in 46 patients (7%) with non-t-AML, with CR/CRi rates of 87 and 84%, respectively.

**Table 2:** Treatment outcomes of patients with AML with normal karyotype.

Therapy	t-AML (*n* = 61)	Non-t-AML (*n* = 681)	*P*-value
	*N* (%)	*N* (%)	
**Intensive induction chemotherapy**	16 (26)	355 (52)	**<0.01**
[CR + CRi]	11 (69)	305 (86)	0.05
CR	11 (69)	283 (80)	0.29
CRi	0 (0)	22 (6)	0.3
MLFS	1 (6)	10 (3)	0.42
PR	0 (0)	7 (2)	0.57
No response	3 (19)	25 (7)	0.06
Non evaluable	1 (6)	8 (2)	
Induction death			
30-day mortality	1 (6)	10 (3)	0.52
60-day mortality	4 (25)	14 (5)	**<0.01**
**Low-intensity induction therapy**	45 (74)	326 (48)	**<0.01**
[CR + CRi]	27 (60)	198 (61)	0.92
CR	23 (51)	155 (48)	0.65
CRi	4 (9)	43 (13)	0.41
MLFS	2 (4)	30 (9)	0.28
PR	0 (0)	6 (2)	0.35
No response	14 (31)	80 (25)	0.9
Non evaluable	2 (5)	12 (3)	
Induction death			
30-day mortality	3 (7)	10 (3)	0.11
60-day mortality	7 (16)	26 (8)	0.09
**HSCT in CR1**	9 (15)	151 (22)	0.17

*t-AML* therapy-related acute myeloid leukemia, *CR* complete remission, *CRi* complete remission with incomplete hematological recovery, *PR* partial remission, *MLFS* morphological leukemia-free state, *HMA* hypomethylating agent, *HSCT* hematopoietic stem cell transplantation, *CR1* first complete remission.

Bold values are statistically significant.

The 60-day mortality was significantly higher in patients with t-AML treated with intensive induction (25% versus 5% for non-t-AML, *P* < 0.01) with three out of the four deaths in the t-AML group occurring in remission due to treatment-related complications. In patients treated with low-intensity therapies, there was a non-statistically significant trend toward increased 60-day mortality in those with t-AML (16% versus 8%, *P* = 0.09).

### Relapse and survival outcomes

**Fig. 2:**
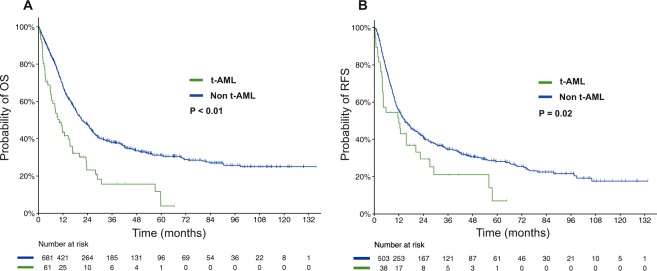
Survival for patients with t-AML with NK and non-t-AML with NK. **a** Overall survival and **b** relapse-free survival of patients with AML and NK.

**Fig. 3:**
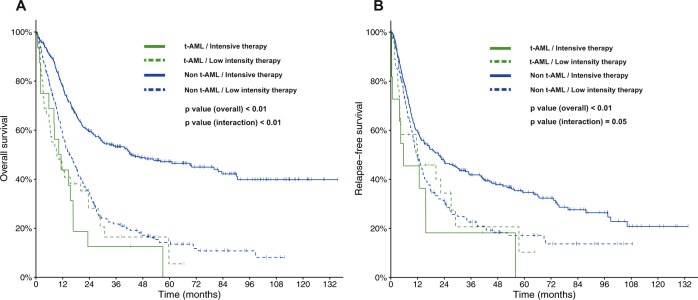
Survival for patients with t-AML with NK and non-t-AML with NK, stratified by treatment intensity (low-intensity versus high-internsity). **a** Overall survival and **b** relapse-free survival, stratified according to treatment intensity.

With a median follow-up of 54.0 months, the median OS and RFS for the entire cohort of patients with AML and NK were 20.3 months and 14.7 months (Supplemental Tables 3 and 4). The estimated 5-year OS and RFS rates were 29% (95% CI, 26%–33%) and 27% (95% CI 23%–32%), respectively. Survival outcomes were significantly inferior for patients with t-AML and NK compared to those with non-t-AML and NK. The median OS was 10.3 months and 21.3 months (HR 2.07, 95% CI, 1.54–2.78, *P* < 0.01), and the median RFS was 12.0 months and 14.9 months (HR 1.55, 95% CI 1.06–2.26, *P* = 0.02) for t-AML and non-t-AML, respectively (Fig. 2). Importantly, there was a statistically significant interaction between type of AML and intensity of therapy for OS (*P* < 0.01) and RFS (*P* = 0.05). Accordingly, intensive induction was only beneficial in patients with non-t-AML with median OS of 43.0 months and 10.8 months for patients receiving high- and low-intensity therapy, respectively (*P* < 0.001), whereas outcomes for patients with t-AML were similarly poor regardless of the intensity of therapy (Fig. 3a). Similar findings were seen for RFS although the interaction was marginally significant (*P* = 0.05) and the benefit of intensive chemotherapy in patients with non-t-AML was less pronounced (Fig. 3b). In multivariate analysis adjusting for age, performance status, white blood cell counts at diagnosis, ELN 2017 risk classification, treatment intensity, and HSCT in CR1, t-AML was not independently associated with OS (HR 1.60, 95% CI 0.96–2.65, *P* = 0.07) or RFS (HR 1.55, 95% CI 0.83–2.87, *P* = 0.17; Table 3).

**Table 3:** Multivariable analyses of overall and relapse-free survival.

	Overall survival		Relapse-free survival	
Variable	HR [95% CI]	*P*-value	HR [95% CI]	*P*-value
t-AML (versus non-t-AML)	1.60 [0.96–2.67]	0.07	1.55 [0.83–2.87]	0.17
Age (continuous)	1.03 [1.01–1.04]	**<0.01**	1.01 [0.99–1.02]	0.43
Performance status (≥2 vs 0-1)	1.25 [0.84–1.86]	0.28	1.16 [0.74–1.82]	0.52
WBC count (continuous)	1.01 [1.00–1.01]	**<0.01**	1.00 [0.99–1.01]	0.21
ELN 2017 adverse risk (versus others)	1.48 [1.10–2.00]	**0.01**	1.34 [0.96–1.86]	0.09
Intensive induction therapy (versus low intensity)	1.08 [0.71–1.66]	0.71	1.02 [0.64–1.62]	0.95
HSCT in CR1 (time dependent)	0.45 [0.28–0.71]	**<0.01**	0.53 [0.35–0.80]	**< 0.01**

*t-AML* therapy-related acute myeloid leukemia, *WBC* white blood cells, *ELN* European LeukemiaNet, *HSCT* hematopoietic stem cell transplantation, *CR1* first complete remission.

Bold values are statistically significant.

**Fig. 4:**
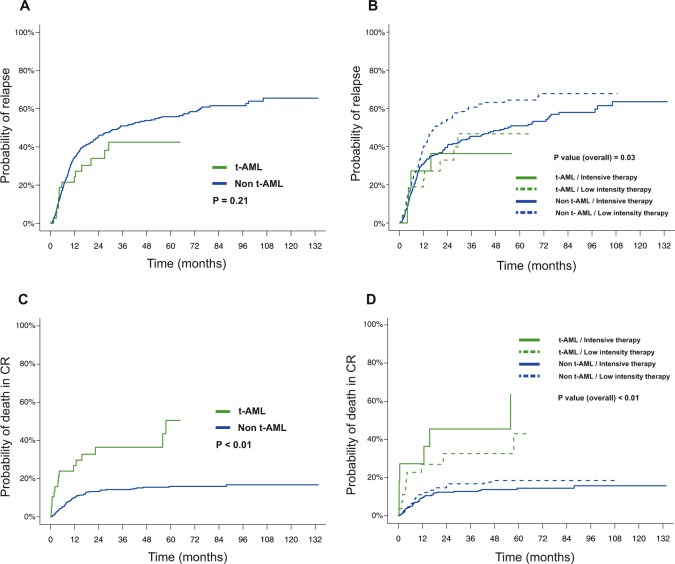
Cumulative incidence of relapse and cumulative incidence of death in remission in patients with t-AML with NK and non-t-AML with NK. **a** Cumulative incidence of relapse, **b** Cumulative incidence of relapse, stratified by treatment intensity (low-intensity versus high-intensity), **c** Cumulative incidence of death in remission, **d** Cumulative incidence of death in remission, stratified by treatment intensity (low-intensity versus high-intensity).

The 5-year CIR rate was similar for patients with t-AML with NK and non-t-AML with NK (42% versus 56%, *P* = 0.21, Fig. 4a). When stratified according to induction intensity, the CIR was the highest among patients with non-t-AML treated with low intensity therapy and the CIR was similar among patients with t-AML regardless of induction intensity (*P* = 0.03 for comparison for all 4 groups; Fig. 4b). In contrast, the 5-year CID was significantly higher in patients with t-AML compared to patients with non-t-AML (51% versus 16%, *P* < 0.01, Fig. 4c), regardless of treatment intensity (Fig. 4d). Results were similar in sensitivity analyses with censoring at time of HSCT in CR1 with no statistically significant difference in CIR, but increased risk of death in CR for patients with t-AML (Supplemental Figs. 1–2). In univariate analysis, t-AML and age >60 were associated with higher CID (*P* < 0.01 and 0.03, respectively) but not CIR (*P* = 0.22 and 0.07, respectively) (Supplemental Table 5). Low intensity induction was associated with higher CIR (62% vs 50%, *P* = 0.02), but not with CID (*P* = 0.2). These findings suggest that non-relapse mortality was the main driver for worse outcomes in patients with t-AML and NK compared to those with non-t-AML and NK. The distribution of causes of deaths in remission was comparable between groups (Supplemental Table 6). Among the 14 deaths in CR in the t-AML subgroup, the three main etiologies of death were: infection, HSCT-related toxicity and concurrent second malignancy, occurring in two patients (14%) each. Additionally, 60% of deaths in CR in patients with t-AML occurred while still on therapy or within 8 weeks of last dose.

### Benefit of allogeneic hematopoietic stem cell transplantation

HSCT in CR1 was associated with improved OS (HR 0.48, 95% CI 0.37–0.64, *P* < 0.01) and RFS (HR 0.57, 95% CI 0.44–0.74, *P* < 0.01) in the overall population when HSCT in CR1 was considered a time-dependent variable. However, the benefit of HSCT in CR1 on OS and RFS was restricted to patients with non-t-AML (HR 0.47, 95% CI 0.53–0.62, *P* < 0.01 for OS; HR 0.55, 95% CI 0.42–0.72, *P* < 0.01 for RFS), whereas patients with t-AML did not derive any benefit from HSCT in CR1 (HR 0.94, 95% CI 0.38–2.33, *P* = 0.89 for OS; HR 1.34, 95% CI 0.52–3.44, *P* = 0.55 for RFS) (Supplemental Table 7). Among patients who proceeded to HSCT in CR1, post-HSCT OS and RFS were significantly worse in patients with t-AML with no patient surviving beyond 5 years, although this analysis is limited by the small number of patients (Supplemental Fig. 3). The CIR after HSCT was similar between patients with t-AML and NK or non-t-AML with NK whereas the CID was significantly increased in patients with NK (Supplemental Fig. 4).

## Discussion

To our knowledge, this study represents the largest comprehensive evaluation of t-AML with NK. We show that t-AML with NK is a rare entity, accounting for 3% of all AML and 17% of t-AML, and is associated with inferior outcome compared with non-t-AML with NK, with a two-fold increase in the risk of death, and significantly shorter median OS (10.3 versus 20.3 months) and RFS (12.0 versus 14.9 months). Importantly, we show that the inferior outcome was largely driven by an increased risk of death in remission rather than differences in molecular features or relapse risk.

Within this subset of patients with AML, we confirm that factors other than cytogenetics account for the adverse prognosis of patients with t-AML and NK. We investigated the mutational profiles among our cohort of patients with NK to evaluate whether adverse molecular features in t-AML could explain differences in prognosis. Interestingly, no significant difference was noted in the mutational frequencies or ELN 2017 risk category between the two groups. The frequencies of *NPM1* (39–45%) and *FLT3-*ITD (23–30%) mutations were similar in both groups and comparable to what has been reported in large datasets of AML with NK^3,16^. Patients with t-AML had a slightly higher frequency of *TP53* mutation (9% versus 4%) and lower frequency of *CEBPA* mutations (5% versus 15%), although these differences were not statistically significant, possibly due to the small numbers of sequenced patients in the t-AML subgroup. Therefore, these data suggest that t-AML with NK often has similar disease biology to non-t-AML, at least as reflected by the presence of recurrent molecular mutations. The similar mutational spectra observed in patients with t-AML and non-t-AML with NK question the pathogenic role of prior chemotherapy or radiotherapy in the development of NK AML. Thus, in the rare subset of patients with chemotherapy or radiation exposure who later develop NK AML, our data suggest that the association between prior therapy and AML diagnosis may be coincidental rather than causative.

Remission rates and relapse rates were also similar between patients with t-AML or non-t-AML with NK, which provides further support that prior exposure to chemotherapy or radiation, per se, may not necessarily lead to a more aggressive disease biology. These findings are consistent with prior data that have linked poor response to therapy among t-AML largely to specific adverse chromosomal abnormalities, which are generally enriched in this population^21^. Nonetheless, post-remission outcomes were significantly different between groups. t-AML had significantly higher 60-day mortality, especially when treated with intensive induction therapy. Notably, deaths in remission were mostly due to infections and there was no substantial contribution from a concurrent malignancy other than AML. Indeed, survival of t-AML with NK was significantly inferior compared with non-t-AML with NK. Although multivariate analysis showed that t-AML was not an independent factor for RFS, there was a trend toward statistical significance for OS (HR 1.6, *P* = 0.07). Importantly, we show that the poorer RFS and OS in patients with t-AML and NK are due to a markedly higher CID (51% versus 16%, *P* < 0.01) rather than an increased risk of relapse. This increased CID among patients with t-AML was observed in both those who received low-intensity therapy and those who received high-intensity therapy, and both when we censored survival at the time of HSCT and when we considered post-HSCT outcomes in patients who proceeded to HSCT in CR1. Although this might suggest that intensive induction is not associated with any benefit in t-AML with normal karyotype, these findings have limitations. Firstly, this is a retrospective study and interpretation of such analysis must be done with caution, especially since we did not take into account many patient-related factors that may confound the results. Secondly, most patients with t-AML were older than 60 years, and therefore these findings may not be applicable to younger patients.

Most prior studies reporting on the inferior survival of t-AML have not evaluated CIR and CID, therefore limiting our ability to understand whether the adverse outcome of t-AML is due to more aggressive disease biology or patient-related factors, including tolerance of therapy. Notably, one group previously showed that the outcome of t-AML varied according to age, and demonstrated that t-AML was associated with increased CID but not CIR in younger patients treated with intensive chemotherapy^3^. However, in older patients, they observed that t-AML was associated with an increased CIR but not with an increased CID, which contrasts with our findings. They suggested that the higher risk of relapse in older patients with t-AML may be explained by the administration of lower-intensity therapy, but it could also be attributed to the higher frequency of adverse features in older patients with AML. However, it is important to note that, in our cohort, most patients with t-AML were ≥ 60 years of age (90%). In addition, our analysis accounted for therapy intensity, and showed that the CIR among patients with NK t-AML was similar to that of patients with non-t-AML treated with intensive therapy, whereas CID was significantly higher for patients with NK t-AML, regardless of therapy intensity. Intensity of therapy was not associated with CID and the difference in CID was relatively modest when patients were stratified by age (22% versus 14%, *P* = 0.03). In contrast, the rate of CID was over 3-fold higher in patients with t-AML versus those with non-t-AML (51% versus 16%, *P* < 0.01), further suggesting that prior exposure to chemotherapy or radiation is the dominant factor driving CID and thus inferior survival.

HSCT in CR1 has been demonstrated to reduce the risk of relapse in subsets of patients with AML and NK^22–25^. Among all patients with NK in our cohort, HSCT in CR1 was associated with improved OS and RFS. However, this benefit was restricted to patients with non-t-AML. The 4-year non-relapse mortality after transplantation was about 40% in patients with t-AML, similar to previous reports on t-AML, which likely outweighs the potential benefit of HSCT in most of these patients^3,26^. Our data therefore suggest that HSCT in CR1 likely offers limited benefit in most patients with NK t-AML because of a high rate of death in remission. However, it is important to note that 90% of patients with t-AML and NK in our dataset were ≥60 years of age, and therefore we cannot extrapolate these data to the rare patients who are younger than 60 years of age. Proceeding to HSCT in CR1 may still be beneficial to these patients if they are fit and otherwise meet the indications for HSCT.

These findings have significant clinical implications. With similar mutation profiles, response to therapy and risk of relapse between NK t-AML and non-t-AML, our study suggests that the adverse outcome observed in patients with NK t-AML is mostly driven by poorer tolerance to therapy rather than more aggressive disease biology. We hypothesize that older age, comorbid medical conditions, sequelae of prior disease, and the cumulative toxicity of primary and secondary cancer therapy may all contribute to this higher non-relapse mortality rate. Since prior reports have consistently shown poor OS and RFS with high rate of relapse in patients with t-AML, the general practice has been to treat these patients with intensive induction therapy followed by consolidation with HSCT in CR1. Based on our findings, even though t-AML with NK has similar disease biology as non-t-AML with NK, a comprehensive evaluation of patients’ comorbidities and fitness level is particularly imperative in patients with t-AML in order to appropriately select therapy as high rates of death in remission are observed in these patients, particularly with intensive therapy. Consequently, the threshold to consider a patient eligible to receive intensive chemotherapy and undergo HSCT in CR1 may need to be higher in patients with NK t-AML. This might be particularly relevant considering the improved outcomes with lower-intensity regimens such as the combination of hypomethylating agents and venetoclax for the treatment of older patients or patients deemed ineligible for intensive chemotherapy^27,28^.

In conclusion, we have shown that, among patients with NK, prior exposure to chemotherapy or radiation is associated with poorer outcomes because of a higher risk of death in remission rather than an increased risk of relapse. Although disease biology appears similar between t-AML and non-t-AML with NK, careful therapeutic decision-making is required to mitigate potential treatment-related toxicity for patients with t-AML and NK.

## Supplementary information

Supplemental Material

Reproducibility checklist

## Data Availability

Supplemental materials are attached.
