# Supplementary material for: Characteristics and outcomes of patients with therapy-related acute myeloid leukemia with normal karyotype
Source: Blood Cancer J. 2020 May 4;10(5):47. doi: 10.1038/s41408-020-0316-3 (PMC7198507; doi:10.1038/s41408-020-0316-3)
Supplement: Supplementary file 1 — Supplemental Material [file 41408_2020_316_MOESM1_ESM.docx]

**Characteristics and Outcomes of Therapy-Related Acute Myeloid Leukemia with Normal Karyotype**

Supplemental Material

**Supplemental Table 1.** Induction regimens

**Supplemental Table 2.** Antecedent hematologic disorder for patients with AML and normal karyotype

**Supplemental Table 3.** Overall survival

**Supplemental Table 4**. Relapse-free survival

**Supplemental Table 5.** Univariate analyses for cumulative incidence of relapse and cumulative incidence of death

**Supplemental Table 6.** Causes of death in first remission

**Supplemental Table 7.** Impact of HSCT on overall and relapse-free survival stratified by t-AML vs non t-AML

**Supplemental Figure 1.** (A) Overall and (B) relapse-free survival censored at transplant

**Supplemental Figure 2.** (A) Cumulative incidence of relapse and (B) of death in remission censored at transplant

**Supplemental Figure 3.** (A) Overall and (B) relapse-free survival post-transplant

**Supplemental Figure 4.** (A) Cumulative incidence of relapse and (B) of death in remission post-transplant

**Supplemental Table 1. Induction treatment regimens of patients with AML and NK (t-AML and non t-AML)**

| **Treatment** | **t-AML (n=61)** | **Non t-AML (n=681)** |
| --- | --- | --- |
| **Intensive regimens** | **N=16 (%)** | **N=355 (%)** |
| IA-based | 8 (50) | 118 (33) |
| BIDFA | 3 (19) | 16 (5) |
| CIA-based | 2 (13) | 72 (20) |
| CLIA-based | 1 (6) | 75 (21) |
| 7+3-based | 1 (6) | 18 (5) |
| CPX-351-based | 1 (6) | 5 (1) |
| FIA-based | 0 | 44 (13) |
| FLAG-IDA | 0 | 7 (2) |
| **Low intensity regimens** | **N=45 (%)** | **N=326 (%)** |
| HMA-based (non Venetoclax) | 16 (36) | 110 (34) |
| LDAC-based | 13 (29) | 98 (30) |
| HMA + Venetoclax | 8 (18) | 46 (14) |
| HMA alone | 6 (13) | 30 (9) |
| Others | 2 (4) | 42 (13) |

Abbreviations: t-AML; therapy-related acute myeloid leukemia, NK; normal karyotype, IA; idarubicin + cytarabine (intermediate-high dose), CIA; clofarabine + idarubicin + cytarabine, FIA; fludarabine + idarubicin + cytarabine, CLIA; cladribine + idarubicin + cytarabine, 7+3; standard dose cytarabine + anthracycline, FLAG-IDA; fludarabine + cytarabine + idarubicin + G-CSF, BIDFA; fludarabine + cytarabine twice daily, HMA; hypomethylating agent, LDAC; low dose cytarabine.

**Supplemental Table 2. Antecedent hematologic disorder for patients with AML and NK**

| **Prior myeloid neoplasm** | **t-AML** | **Non t-AML** |
| --- | --- | --- |
| MDS | 5 | 41 |
| MPN | 0 | 22 |
| MDS/MPN | 0 | 5 |
| CMML | 1 | 13 |
| **Total** | **6** | **80** |

Abbreviations: t-AML; therapy-related acute myeloid leukemia, MDS; myelodysplastic syndrome, MPN; myeloproliferative neoplasm, CMML; chronic myelomonocytic neoplasm

**Supplemental Table 3. Overall survival of patients with AML and NK (t-AML and non t-AML)**

|  | | **Overall population**  **(N = 742)** | **t-AML**  **(N = 61)** | **Non t-AML**  **(N = 681)** | **P** |
| --- | --- | --- | --- | --- | --- |
| **All patients** | |  |  |  |  |
| Median OS | | 20.3 m [18.2 – 23.6] | 10.3 m [6.5 – 16.7] | 21.3 m [18.9 – 25.1] | **< 0.001** |
| 3-year OS | | 36.3% [32.7 – 40.3] | 15.7% [8.0 – 30.6] | 38.1% [34.3 – 42.3] |  |
| 5-year OS | | 29.1% [25.5 – 33.1] | 3.9% [0.6 – 24.3] | 31.2% [27.5 – 24.3] |  |
|  | |  |  |  |  |
| **Intensive therapy** |  |  |  |  |  |
| Median OS | | 41.3 m [28.4 – 69.2] | 10.8 m [5.8 – 23.6] | 43.0 m [29.8 – 81.9] | **< 0.001** |
| 3-year OS | | 51.3% [46.1 – 57.1] | 12.5% [3.4 – 45.7] | 53.2% [47.9 – 59.1] |  |
| 5-year OS | | 44.9% [39.5 – 51.0] | NA | 47.1% [41.6 – 53.4] |  |
|  | |  |  |  |  |
| **Low intensity therapy** | |  |  |  |  |
| Median OS | | 14.2 m [12.4 – 17.6] | 9.5 m [6.0 – 23.6] | 15.3 m [13.0 – 18.5] | 0.13 |
| 3-year OS | | 21.1% [17.0 – 26.3] | 16.4% [7.3 – 36.9] | 21.7% [17.3 – 27.2] |  |
| 5-year OS | | 13.2% [9.6 – 18.1] | 5.5% [0.9 – 32.9] | 14.2% [10.3 – 19.5] |  |

Abbreviations: t-AML; therapy-related acute myeloid leukemia, NK; normal karyotype, OS; overall survival, RFS; relapse-free survival

**Supplemental Table 4. Relapse-free survival of patients with AML and NK (t-AML and non t-AML)**

|  | **Overall population**  **(N = 742)** | | **t-AML**  **(N = 61)** | **Non t-AML**  **(N = 681)** | **P** |
| --- | --- | --- | --- | --- | --- |
| **All patients** |  | |  |  |  |
| Median RFS | 14.7 m [12.5 – 17.8] | | 12.0 m [4.3 – 27.1] | 14.9 m [12.7 – 19.3] | 0.02 |
| 3-year RFS | 33.9% [29.8 – 38.5] | | 21.1% [10.4 – 42.8] | 34.7% [30.5 – 39.5] |  |
| 5-year RFS | 26.9% [22.9 – 31.6] | | 7.0% [1.2 – 40.4] | 28.2% [24.0 – 33.1] |  |
|  |  | |  |  |  |
| **Intensive therapy** |  | |  |  |  |
| Median RFS | 19.5 m [13.8 – 29.5] | | 5.7 m [3.9 – NA] | 21.5 m [14.9 – 30.9] | 0.007 |
| 3-year RFS | 41.0% [35.6 – 47.1] | | 18.2% [5.2 – 63.7] | 41.8% [36.4 – 48.1] |  |
| 5-year RFS | 33.4% [28.0 – 39.9] | | NA | 34.7% [29.1 – 41.3] |  |
|  |  | |  |  |  |
| **Low intensity therapy** | |  |  |  |  |
| Median RFS | 11.9 m [10.2 – 14.5] | | 12.2 m [4.3 – NA] | 11.5 m [10.0 – 14.7] | 0.85 |
| 3-year RFS | 22.6% [17.1 – 29.8] | | 20.6% [8.2 – 51.9] | 22.6% [16.9 – 30.2] |  |
| 5-year RFS | 16.4% [11.4 – 23.7] | | 10.3% [2.0 – 54.5] | 17.1% [11.8 – 24.8] |  |

Abbreviations: t-AML; therapy-related acute myeloid leukemia, NK; normal karyotype, OS; overall survival, RFS; relapse-free survival

**Supplemental Table 5. Univariate analyses for cumulative incidence of relapse (CIR) and cumulative incidence of death (CID)**

| **Variable** | **5-year CIR**  **% [95% CI]** | **CIR**  **P value** | **5-year CID**  **% [95% CI]** | **CID**  **P value** |
| --- | --- | --- | --- | --- |
| **Therapy-related AML** |  |  |  |  |
| t-AML | 42 [24 – 61] | 0.22 | 51 [28 – 73] | **< 0.01** |
| Non t-AML | 55 [51 – 61] |  | 15 [12 – 19] |  |
| **Age at diagnosis** |  |  |  |  |
| Age < 60 y.o. | 50 [43 – 57] | **0.07** | 14 [9 – 19] | **0.03** |
| Age ≥ 60 y.o. | 59 [53 – 66] |  | 22 [17 – 27] |  |
| **Performance status** |  |  |  |  |
| ECOG 0-1 | 57 [51 – 62] | 0.59 | 17 [13 – 21] | 0.17 |
| ECOG 2 | 54 [41 – 67] |  | 24 [13 – 35] |  |
| **WBC at diagnosis** |  |  |  |  |
| WBC < 100 x 10^9^/L | 55 [50 – 59] | 0.58 | 18 [14 – 21] | 0.31 |
| WBC ≥ 100 x 10^9^/L | NA |  | NA |  |
| **Hb at diagnosis** |  |  |  |  |
| Hb < 10 g/dL | 56 [51 – 62] | 0.71 | 19 [15 – 24] | 0.30 |
| Hb ≥ 10 g/dL | 51 [43 – 60] |  | 15 [9 – 21] |  |
| **Plt at diagnosis** |  |  |  |  |
| Platelets < 100 x 10^9^/L | 56 [51 – 61] | 0.39 | 19 [15 – 23] | 0.46 |
| Platelets ≥ 100 x 10^9^/L | 51 [41 – 62] |  | 16 [8 – 23] |  |
| **ELN 2017 risk classification** |  |  |  |  |
| Favorable | 46 [32 – 60] | 0.62 | 9 [5 – 16] | **0.04** |
| Intermediate | 51 [36 – 66] |  | 21 [8 – 34] |  |
| Adverse | 40 [27 – 52] |  | 28 [18 – 39] |  |
| **Therapy intensity** |  |  |  |  |
| Low intensity | 62 [55 – 70] | **0.02** | 21 [15 – 27] | 0.20 |
| High intensity | 50 [44 – 56] |  | 16 [12 – 21] |  |
| **NPM1 mutation** |  |  |  |  |
| *NPM1* wild-type | 63 [57 – 70] | **< 0.01** | 17 [12 – 21] | 0.33 |
| *NPM1* mutation | 42 [35 – 49] |  | 22 [16 – 29] |  |
| ***FLT3* mutation** |  |  |  |  |
| *FLT3* wild-type | 57 [51 – 63] | 0.25 | 14 [10 – 18] | **< 0.01** |
| *FLT3-*ITD mutation | 50 [42 – 58] |  | 25 [18 – 33] |  |
| ***RUNX1* mutation** |  |  |  |  |
| *RUNX1* wild-type | 48 [40 – 56] | 0.91 | 20 [12 – 28] | 0.29 |
| *RUNX1* mutation | NA |  | NA |  |
| ***ASXL1* mutation** |  |  |  |  |
| *ASXL1* wild-type | 49 [39 – 59] | 0.44 | 20 [11 – 28] | 0.15 |
| *ASXL1* mutation | 37 [22 – 51] |  | 26 [14 – 39] |  |
| ***TP53* mutation** |  |  |  |  |
| *TP53* wild-type | 46 [39 – 54] | 0.55 | 20 [14 – 26] | 0.06 |
| *TP53* mutation | NA |  | NA |  |
| ***CEBPA* mutation** |  |  |  |  |
| *CEBPA* wild-type | 52 [46 – 59] | 0.63 | 20 [15 – 26] | 0.40 |
| *CEBPA* mutation | 51 [37 – 65] |  | 14 [5 – 24] |  |

**Supplemental Table 6.** **Causes of death in first remission**

| **Cause of Death** | **t-AML (N=61)** | **Non t-AML (N=681)** |
| --- | --- | --- |
| Infection | 2 (3) | 23 (3) |
| HSCT related-toxicity | 2 (3) | 21 (3) |
| Second primary Cancer | 2 (3) | 2 (1) |
| Bleeding | 1 (2) | 0 |
| Cardiac/dementia/cirrhosis/stroke | 1 (2) | 4 (1) |
| Unknown | 6 (10) | 20 (3) |
| **Total** | **14 (23)** | **75 (11)** |

Abbreviations: t-AML; therapy-related acute myeloid leukemia, HSCT: hematopoietic stem cell transplant

**Supplemental Table 7. Impact of HSCT on Overall and relapse-free survival stratified by t-AML vs. non t-AML**

|  | **Overall survival** | | **Relapse-free survival** | |
| --- | --- | --- | --- | --- |
| **Variable** | **HR [95% CI]** | **P** | **HR [95% CI]** | **P** |
| t-AML | 0.94 [0.38 – 2.33] | 0.89 | 1.34 [0.52 – 3.44] | 0.55 |
| Non t-AML | 0.47 [0.53 – 0.62] | **< 0.01** | 0.55 [0.42 – 0.72] | **< 0.01** |

Abbreviations: HSCT: hematopoietic stem cell transplant, t-AML; therapy-related acute myeloid leukemia

**Supplemental Figure 1.** **(A) Overall and (B) relapse-free survival censored at transplant**


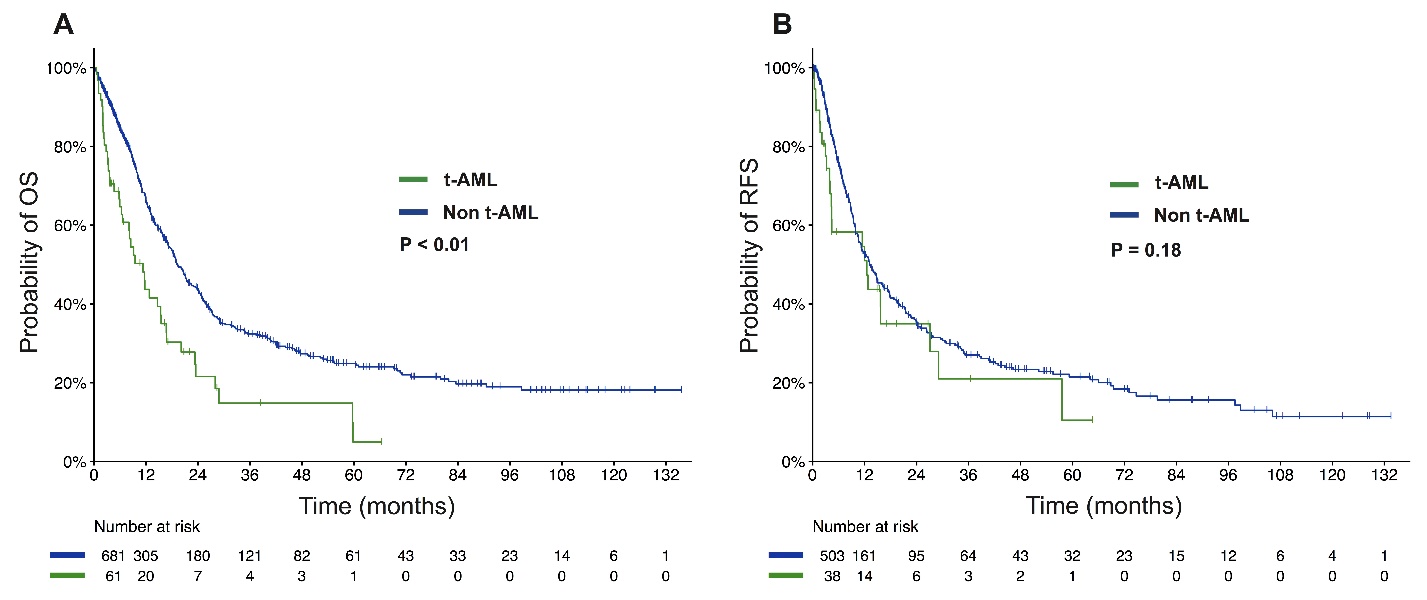


**Supplemental Figure 2. (A) Cumulative incidence of relapse and (B) of death in remission censored at transplant**

**
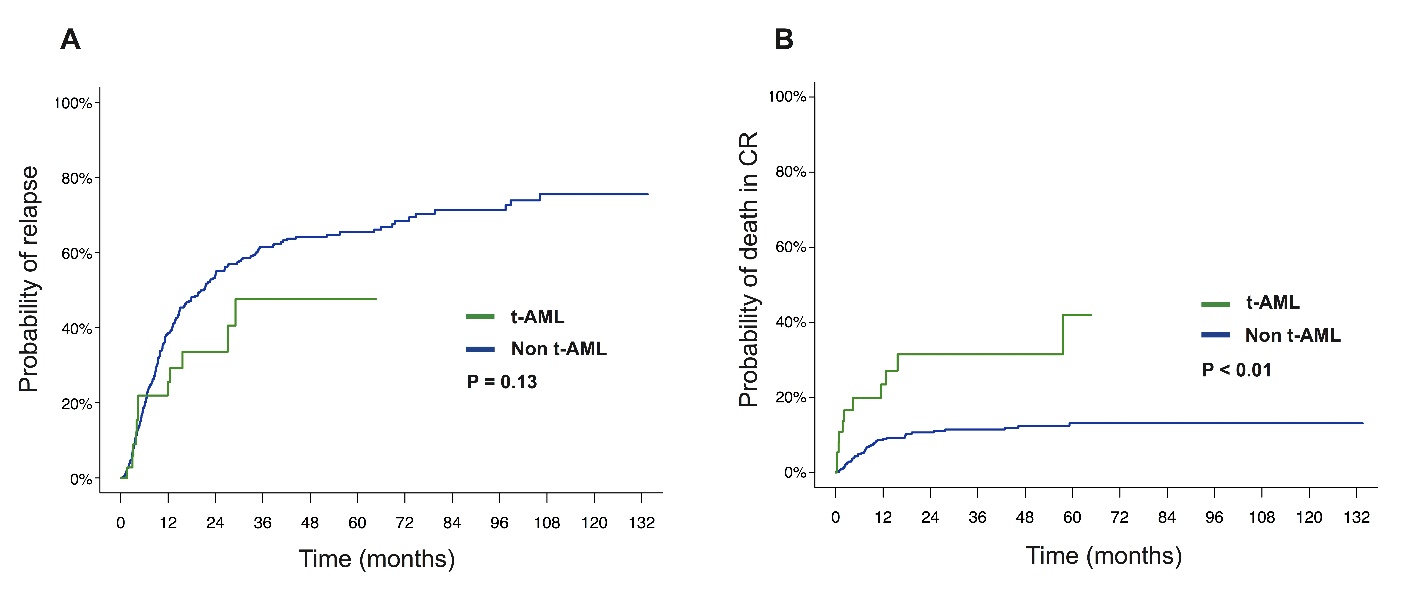
**

**Supplemental Figure 3.** **(A) Overall and (B) relapse-free survival post-transplant**

**
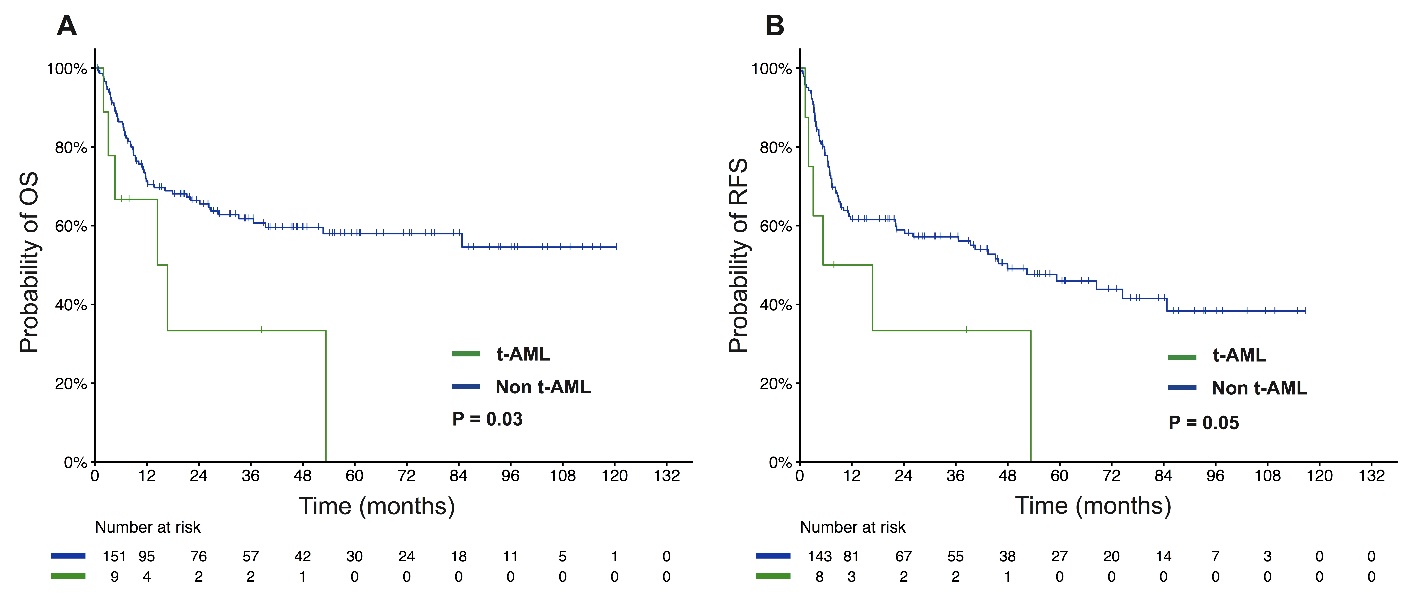
**

**Supplemental Figure 4.** **(A) Cumulative incidence of relapse and (B) of death in remission post-transplant**

**
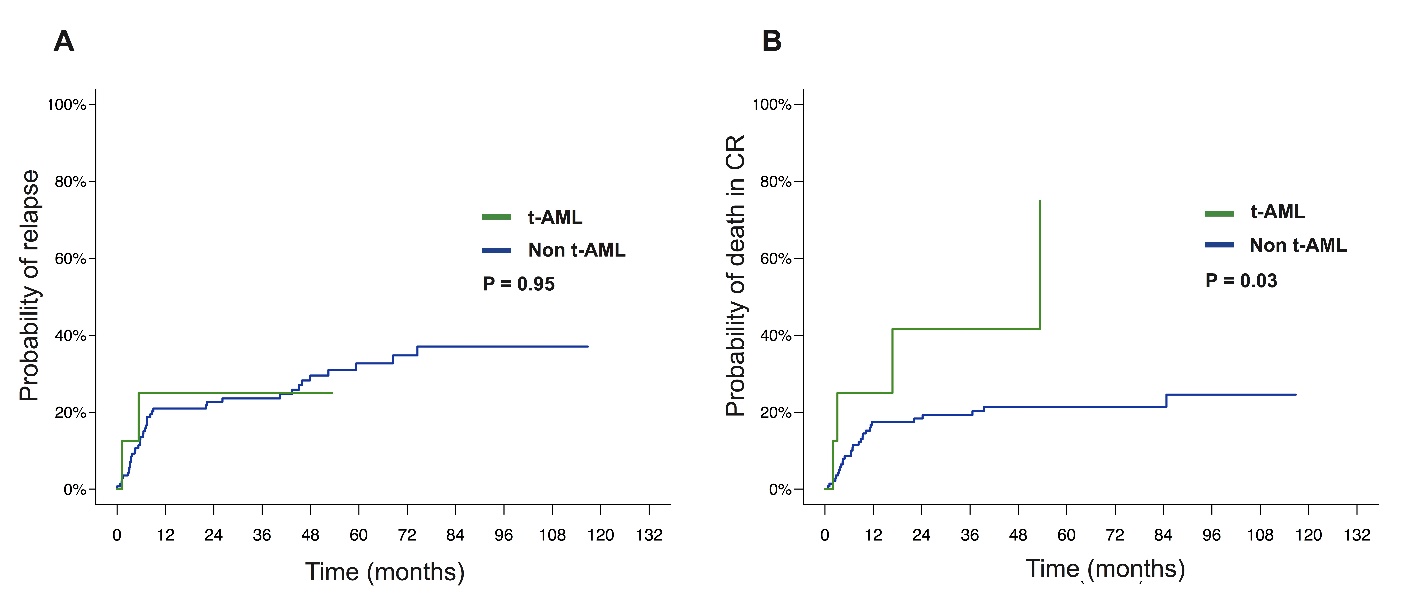
**
